# Multiplexing of TMT labeling reveals folate-deficient diet-specific proteome changes in NTDs

**DOI:** 10.3389/fcell.2024.1294726

**Published:** 2024-03-13

**Authors:** Pei Pei, Jinying Shen, Xuejia He, Yubing Zeng, Ting Zhang, Shan Wang

**Affiliations:** ^1^ Beijing Municipal Key Laboratory of Child Development and Nutriomics, Capital Institute of Pediatrics, Beijing, China; ^2^ Capital Institute of Pediatrics-Peking University Teaching Hospital, Beijing, China; ^3^ Children’s Hospital Capital Institute of Pdiatrics, Chinese Academy of Medical Sciences and Peking Union Medical College, Beijing, China

**Keywords:** neural tube defects, proteomics, folate deficiency, ribosome, RPL13, RPL14, neural tube development

## Abstract

**Introduction:** In the early stage of embryonic development, the neural tube (NT) cannot be closed properly due to some complex factors, including environmental factors, genetic factors, and the relationship between various factors, leading to the occurrence of neural tube defects (NTDs).

**Methods:** In this study, we induced a mouse model of NTDs by feeding mice with a low-folate diet and intraperitoneally injecting them with 1.5 mg/kg methotrexate on E7.5. Fetal mice were achieved at E13.5, and we extracted proteins from brain tissues with trypsin digestion. After enzymatic digestion, peptides were labeled with TMT/iTRAQ and separated in high-performance liquid chromatography (HPLC) for subsequent liquid chromatography tandem mass spectroscopy (LC-MS/MS) analysis. We used gene ontology (GO) and Kyoto Encyclopedia of Genes and Genomes (KEGG) pathway annotation to analyze proteomic changes and analyze the functional enrichment of differentially expressed proteins (DEPs) in the NTD mice tissues.

**Results:** A low-folate-induced mouse model was successfully constructed. Folate was used as a sensitizing agent, and the teratogenicity rate of the NTD fetal mice increased to 36.5% when the concentration of methotrexate was at 1.5 mg/kg. Mass spectrometry was used to identify 6,614 proteins, and among them, 5,656 proteins were quantified. In the following proteomic analysis, GO classification and KEGG pathway enrichment analysis were conducted, and heatmaps were drawn for differentially expressed proteins (DEPs). The main pathways associated with NTDs, such as the Hedgehog, Wnt, p53, and Hippo signaling pathways and the one-carbon pool mediated by folate, can be identified through a protein–protein interaction (PPI) network. It was also found that the regulation of ribosomal proteins, such as RPL13 and RPL14, which are upregulated in NTDs, has a certain impact on neural tube development.

**Discussion:** Our results revealed proteomic changes in the tissues of low-folate-induced NTD mice. Validation showed that ribosomal proteins play a regulatory role during the development of NTDs and provides new ideas for the pathogenesis and preventive measures of NTDs.

## 1 Introduction

Neural tube defects (NTDs) are severe congenital abnormalities of the central nervous system that are caused by abnormal closure of the neural tube during embryonic development. The central nervous system (CNS) develops from the neural tube (NT), which mainly includes the spinal cord and the brain (Finnell et al., 2002). The earliest stage of CNS development is neural induction, in which ectodermal cells adopt the principles of neural properties (Copp et al., 2003). Shaping, bending, and fusion with the neural plate form the neural tube in higher vertebrates. During the process of neural tube generation, the fusion of the dorsal midline gradually seals the neural tube. Neurological defects and degeneration are likely to occur if the neuroepithelium is constantly exposed to the environment due to the inability of the neural tube to close normally (Greene and Copp, 2014). Failure to develop the neural tube results in a cranial fissure, with the entire spine and most of the brain being open. The defect is called anencephaly when the cranial region is defective; spina bifida occurs when the defect is confined to the lumbosacral region (Northrup and KAJCpip, 2000). Therefore, open NTDs cause spina bifida and anencephaly.

Although there is a lack of actual data on NTDs worldwide, an estimated 260,100 cases of NTDs were reported in 2015 (Blencowe et al., 2018). The incidence of NTDs has declined in developed countries, but it remained high in developing countries (Moore et al., 1997). NTDs also have implications for socioeconomic development and various medical issues (Bitew et al., 2020). Among them, known genetic factors, environmental factors, nutritional factors, and the relationship between these factors play a specific role in the occurrence of NTDs (Kondo et al., 2009). Previous research found that a common mutation in 5,10-methylenetetrahydrofolate reductase (MTHFR) was a risk factor for NTDs (Kondo et al., 2009). During the first half of the 20th century, numerous experimental and clinical studies revealed that folic acid (FA) plays a critical role in the development or recurrence of NTDs. FA has been categorized as one of the B vitamins. Earlier research has shown that the administration of folic acid during pregnancy can decrease fetal NTDs by 70% (Lancet, 1991). According to Hibbard’s investigation and experiments, lack of FA supplementation during pregnancy can lead to fetal congenital malformations. Supplementation of FA at the appropriate time before conception is necessary to prevent fetal malformations (Hibbard and Survey, 1965). Although NTDs were very common worldwide and a global public health burden a few decades ago, preventive measures such as folic acid supplementation during pregnancy have been implemented effectively to avoid NTDs (Crider et al., 2011).

Previous research has found that in axons far from the neuron’s cell body, messenger RNAs (mRNAs) encoding many ribosomal proteins (RPs) are powerfully enriched and translated (Shigeoka et al., 2019). In order for mRNA to be properly initiated and translated into proteins, ribosomal proteins, which are major components of protein synthesis, must undergo multiple highly regulated processes to form numerous proteins of the ribosomal subunit. In eukaryotes, ribosomal RNAs (rRNAs) are the most actively transcribed genes, accounting for 80% of the total cellular RNA transcription (Moss et al., 2007). The newly synthesized ribosomes determine the regulation of cell growth. Deregulation of ribosome biogenesis is related to many types of cancer. Among them, Rpl13, Rpl14, and Rpl18A all exist in the 60s ribosomal subunit, and previous studies have shown that they are tumor suppressor genes (Oh et al., 2002). Genuth et al. observed that functional loss of ribosomal protein RPL10A causes an early pronounced mesoderm phenotype in mice and inhibits paraxial mesoderm production (Genuth et al., 2022). In consequence, changes in ribosome expression during development may alter the synthesis of new ribosomes required for cell growth and proliferation (Wlodarczyk et al., 2006). If cell growth and proliferation are delayed during fetal NT closure, the severity of NTDs in embryonic development may be increased by these changes.

Folate is an essential micronutrient throughout life, especially during the early stages of embryonic development. Maintaining adequate folic acid levels during pregnancy is important both for the mother’s health and for the developing fetus (Bailey, 2009). Much effort has been made to link folic acid to neurological disorders, cancer, birth defects, and more (Xu and Chen, 2009; Smith et al., 2010; Blom and Yvo, 2011; Duthie, 2011). In our study, the use of folate antagonist methotrexate (MTX) induced the establishment of NTDs in mice fed a low-folate (LF) diet. Previous studies found that rRNA genes could be detected in MTX-treated mouse embryonic stem cells (mESCs), and the DNA breakage sites of RNA genes were increased after MTX treatment (Xie et al., 2017). These results demonstrated that folate is essential for genome stability and that altering folate levels affect DNA double-strand breaks, which in turn leads to the regulation of rRNA transcription during the early stages of fetal development (Xie et al., 2017). Retinoic acid (RA) is a vitamin A derivative that is involved in neurogenesis and patterning of the neural tube. It is an important signaling molecule that can be detected in all vertebrate embryos (Maden et al., 1996). On the 10.5th day of mouse embryonic development, RA can be detected in the CNS at the lowest levels in the forebrain and midbrain; a large amount of RA is concentrated in the spinal cord (Horton and Maden, 1995). Thus, the development of the neural tube in mice is dependent on the crucial role played by RA. RA doses of 28 mg/kg have been shown in previous studies to cause NTDs, such as anencephaly (Zhang et al., 2009). However, in the current study, regulatory changes of ribosomal proteins (rRNA) in the RA-induced neural tube defect mouse model were not specifically demonstrated. On the other hand, mice with folic acid deficiency-induced NTDs showed upregulated expression of rRNA gene transcription, but only in the tissue of spina bifida. Conversely, in anencephaly, the levels of rRNA gene transcription were significantly decreased (Xie et al., 2017). On the basis of this conclusion, the abnormal regulation of rRNA in NTDs can be further verified to reveal the complexity of NTDs.

Proteomics was performed on the low-folic-acid-induced NTD mouse model in this study. The proteins in normal and NTD-mouse tissues were extracted for TMT labeling and liquid chromatography tandem mass spectroscopy (LC-MS/MS) analysis. To profile the features of proteomics in NTD-mouse tissues induced by low folic acid, functional enrichment analysis of DEPs was conducted through GO analysis, KEGG pathway annotation, protein domain and subcellular localization, and finally, a PPI network diagram. Metabolic and signaling pathways are associated with NTDs in the PPI network, among which we found that ribosomes play an essential role in the process of cell growth and development. It is hypothesized that the ribosome, like other related metabolic pathways, undergoes changes in protein levels during the development of NTDs or that disruption of related pathways during embryonic development may lead to NTDs. Therefore, in subsequent experiments, the effect of ribosomal protein on LF-induced NTD mice can be further verified. Our results shed light on protein level changes in LF-induced NTD mice and provide novel insights into the pathogenesis of NTDs. At the same time, preventive measures can be taken to prevent the occurrence of NTDs in the embryonic process and reduce the risk of fetal NTDs.

## 2 Materials and methods

### 2.1 Animals

*C57BL/6* mice were kept in an authorized facility with specific pathogen-free (SPF) conditions and a 12-h light/dark cycle. Over a 4-week period, sexually mature female and male *C57BL/6* mice were fed a low-folate diet, followed by an overnight mating session. The vaginal plug was inspected the following morning, and this day was determined to be E0.5. The NTD model was created on E7.5 by injecting pregnant mice with 1.5 mg/kg MTX (Sigma USA) intraperitoneally; on E13.5, the mice were euthanized and sacrificed through cervical dislocation. Embryos were removed for dissection, and brain and spine tissues were collected and frozen in test tubes (Pei et al., 2019). All procedures related to the disposal of animals were carried out in accordance with the institutional guidelines for the care of laboratory animals.

### 2.2 Protein extraction

The samples were taken out from −80°C. An appropriate amount of tissue samples was weighed and placed in a mortar pre-cooled with liquid nitrogen, gradually adding liquid nitrogen until the samples were fully ground to powder. Each group of samples was treated with a 4× volume of lysis buffer (consisting of 1% protease inhibitor, 50 mM NAM, 3 μM TSA, and 8 M urea). The samples were sonicated using a high-intensity sonicator to lyse proteins and then centrifuged at 12,000 g for 10 min at 4°C to remove cell fragments. The remaining supernatant was transferred to a new centrifuge tube, and a BCA kit was used to determine the protein concentration.

### 2.3 Trypsin digestion

The protein extract was supplemented with dithiothreitol to achieve a concentration of 5 mM, after which it was subjected to reduction at 56°C for 30 min. Iodoacetamide was used to make the concentration 11 mM alkylate at room temperature for 15 min in the dark. Triethylammonium bicarbonate (TEAB, 100 mM) was added to the protein sample to dilute the urea concentration to less than 2 M. Eventually, trypsin was incorporated into the mixture at a trypsin: protein ratio of 1:150 for the initial overnight enzymatic digestion. Afterward, the enzymatic hydrolysis proceeded by adding trypsin at a mass ratio of 1:100 for 4 h.

### 2.4 TMT labeling

After trypsinization, peptides were desalted through Strata X C18 (Phenomenex) and vacuum freeze-dried. The peptides were dissolved in 0.5 M TEAB solution and labeled following the TMT kit instructions. To summarize, a single unit of labeling reagent was defrosted and dissolved in acetonitrile and then blended with the peptide and incubated at room temperature for 2 h. The resultant mixture was concentrated using vacuum centrifugation, followed by desalting and lyophilization.

### 2.5 HPLC fractionation

Peptides were separated by high pH reverse-phase high-performance liquid chromatography (HPLC) using an Agilent 300 Extend C18 column (5 μm particle size, 4.6 mm id, 250 mm length). In summary, peptides were fractionated into 60 parts within a time frame of 60 min by utilizing a pH 9 gradient of 9%–31% acetonitrile. The 14 peptide components were combined and subjected to vacuum freeze-drying for subsequent procedures.

### 2.6 LC-MS/MS analysis

The peptides were dissolved in LC mobile phase A, which comprised 0.1% formic acid and 2% acetonitrile, before being loaded onto 15-cm-long, 75-μm-diameter vessels. In the EASY-nLC 1000 UHPLC system, with a reverse-phase analytical column, the flow rate was maintained at 500 nL/min, the gradient of mobile phase B (0.1% formic acid in 90% acetonitrile) was increased from 9% to 26% in 20 min, from 26% to 38% in 10 min, then to 80% over 4 min and held at 80% for the last 3 min. The separated peptides were injected into the NSI ion source and then coupled online with Q ExactiveTM Plus (Thermo). The ion source voltage was set at 2.0 kV, and the analysis was detected by tandem mass spectrometry (MS/MS). The scanning range of the main mass spectrometer was set to 400–1,500 m/z, and the scanning resolution was set to 70,000; the scanning range of the secondary mass spectrometer was fixed at 100 m/z, and the scanning resolution was set to 17,500. To enhance the utilization of the mass spectrum, the automatic gain control was configured at 5E4, and the maximum injection time was capped at 80 ms, while the signal threshold was established at 63,000 ions/s.

### 2.7 Immunohistochemistry (IHC)

The dissected mouse embryonic tissue was fixed overnight in 4% paraformaldehyde. After the tissue was removed from fixation, it was rinsed with PBS for 1 h, then dehydrated, and fixed in wax solution. The cubes were cut into thin slices with a continuous thickness of 5 μm. The sections were first treated with xylene to remove the paraffin and then thoroughly rehydrated using a mixture of graded alcohol and water. IHC staining for RPL10A, RPL13, RPL14, and RPL18A on brain tissue was performed using the avidin–biotin complex method (Vector Laboratories). Four antibodies were used in a 1:200 dilution at 4°C for 12 h. The levels of RPL10A, RPL13, RPL14, and RPL18A were analyzed by ImageJ.

### 2.8 Western blot (WB)

The blots were incubated overnight with a monoclonal antibody against rabbit RPL13 (1:1,000, Abcam, United Kingdom) and a monoclonal antibody against rabbit RPL14 (1:5000, Solarbio, China) at 4°C, followed by incubation at room temperature for 1 h with an anti-rabbit enzyme-labeled antibody (1:5000, CST, USA). After rinsing for 3 h with stripping buffer, the membrane was incubated with a housekeeping gene (H3, 1:5000, Abcam, United Kingdom). The imprints were prepared with SuperSignal West Pico chemiluminescence substrate (Termo, United States) and quantified using Quantity One software on a densitometer (Bio-Rad, Universal HoodII, United States).

### 2.9 ELISA

The desired slats were removed from the aluminum foil bag after 20-min equilibration at indoor temperature. The antibody was diluted to a protein content of 1–10 μg/mL with coating buffer, and 0.1 mL/well was added to a polystyrene plate, then incubated overnight at 4°C. Blank wells, negative control wells, and positive control wells were set. An equal amount of the sample was added to the coated reaction wells and incubated at 36°C for 1 h. Freshly diluted enzyme-labeled antibody was added to each reaction well, followed by incubation at 36°C for 30 min. A tetramethylbenzidine (TMB) substrate solution was added to color the reaction well, and finally, the addition of 2 M sulfuric acid stopped the reaction. The OD value of each well on the ELISA detector was measured in the blank control well, and the folic acid concentration in both the normal and low folic acid groups was calculated.

### 2.10 Protein annotation methods

In this study, a gene ontology-based pathway analysis was performed using the gene ontology (GO) vocabulary. GO mainly describes three aspects: molecular function, cell composition, and biological process. GO annotations at the proteomic level were obtained from the UniProt-GOA database. KEGG annotation can integrate information that is currently known as protein interaction networks, for instance, the “Pathway” database, the “Gene” database, the “compound and reaction” database, and annotation of protein pathways using the KEGG pathway database. KEGG pathway annotation and GO analysis were utilized to perform protein functional enrichment, cluster analysis, and subcellular localization.

### 2.11 Protein functional enrichment

The DEP was categorized as 1.2 of fold changes. Proteins that were identified served as the background, and a *p*-value less than 0.05 was determined as significant. Additionally, the KEGG enrichment analysis classified these pathways based on the pathway hierarchy classification on the website. The enrichment of DEP functional domains was analyzed using the InterPro database, and the *p*-value of the protein domain unit enrichment was considered significant if the *p*-value was less than 0.05.

### 2.12 Cluster analysis

A cluster analysis was performed using functional enrichment of DEPs in various groups to explore their possible relationships and distinctive roles in specific functions such as GO, the KEGG pathway, and protein domains. First, the enriched functional categories of the used protein groups were compared with the corresponding enrichment *p*-values. Any functional categories that were significantly enriched in at least one proteome were subsequently filtered out (*p* < 0.05). The filtered *p*-value data matrix was logarithmically transformed by −log10, and the transformed data matrix was Z-transformed for each functional classification. Finally, cluster analysis of the dataset was visualized using the heatmap drawn by the function heatmap.2 in the “gplots” R package.

### 2.13 Protein–protein interaction network

The *STRING (v.10.5)* protein network interaction database was used to compare the database numbers of DEPs screened by different controls. Protein interactions with a confidence score greater than 0.7 (i.e., high confidence) were extracted differentially. The “networkD3” tool in the R package was utilized to visualize the analysis of the differential protein interaction network.

### 2.14 Statistical analysis

In this study, *GraphPad Prism 8.0.1* was used for statistical analysis of all the data. The difference in folic acid concentration in the blood of normal-group maternal mice and LF-fed maternal mice with neural tube defects was compared using a t-test. Statistical significance was defined as a *p*-value < 0.05 and indicated as either *p < 0.05 or **p < 0.01.

## 3 Results

### 3.1 Establishment of an NTD mouse model and extraction of sample size for proteomic analysis

**FIGURE 1:**
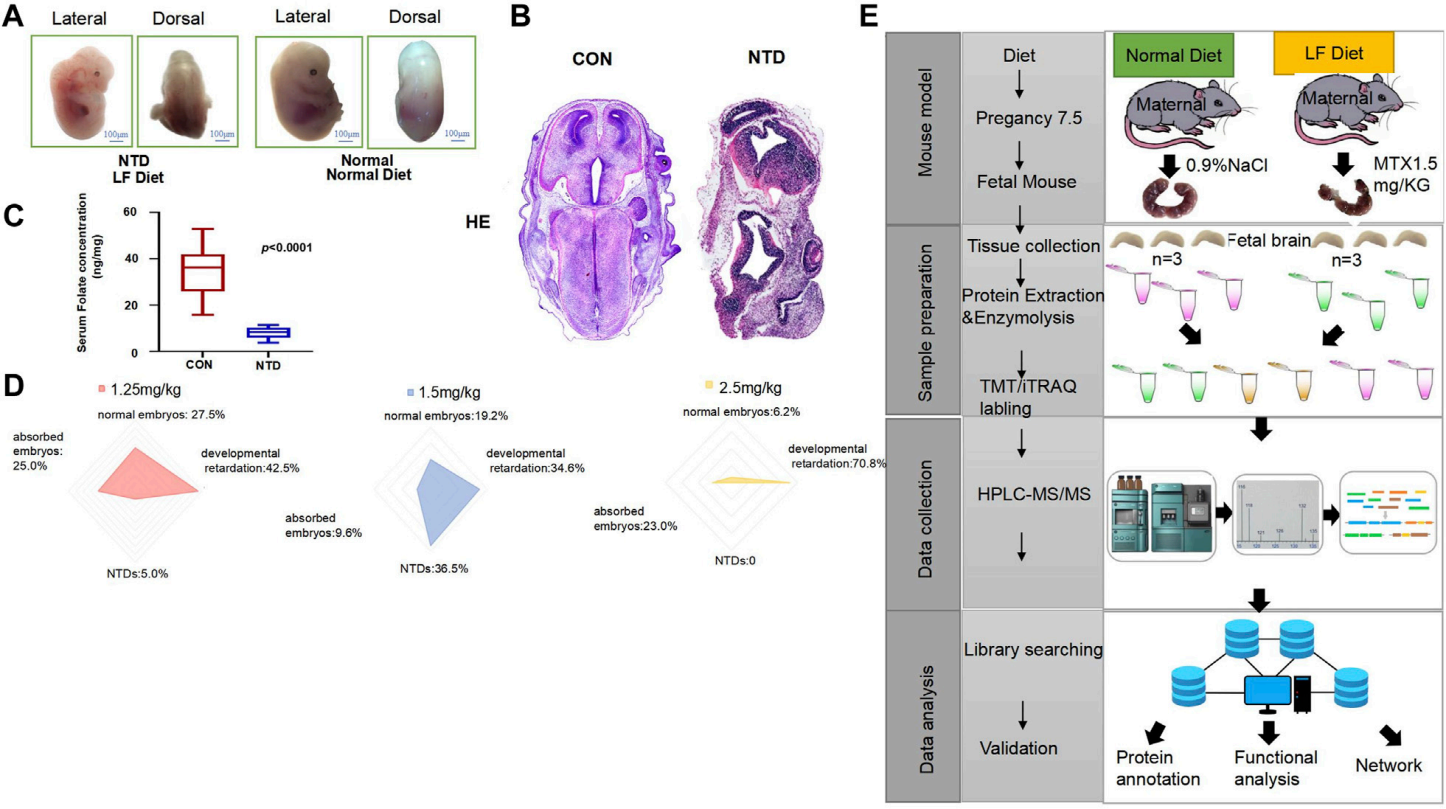
**(A)** Low-folate-diet-induced spina bifida in mouse embryos at 13.5 days. **(B)** Hematoxylin staining of normal and NTD fetal mice. **(C)** Serum folate concentration of female mice in the normal diet and low-folate feeding groups, analyzed by ELISA. Data are mean ± S.D. (*n* = 20). *p < 0.05, by Student’s test. **(D)** The radar chart shows that the embryo phenotypes of the three MTX doses are divided into normal embryos, absorbed embryos, NTDs, and stunted embryos. **(E)** The maternal mice were fed with a normal diet and a low-folate diet at 8 weeks of age for 4 weeks, respectively. Maternal mice under a low-folate diet were injected with 1.5 mg/kgMTX, and the fetal mice were collected at 13.5 days. Fetal neural tissues (brain and spine) were separated from the body in each group, and protein extraction, enzymolysis, and TMT labeling were performed. Samples were separated, and data were collected by HPLC-MS/MS for proteomic analysis.

We designed an experimental method by establishing a low-folate-induced NTD mouse model that allowed us to observe the fetal mice morphology and development of the neural tube at 7.5 days of gestation. It can be clearly seen that the fetal mice fed with a normal diet have a round and smooth shape, a slender tail, and the neural tube is completely closed. Conversely, low-folate-induced NTD mice can be observed with obvious differences in morphology compared with normal fetal mice; failed closure of the neural tubes resulted in exposure of spinal nerves, and the spinal cords exhibited a winding and tapered look (Figure 1A).

In order to clearly observe the morphological differences between the normal group and the NTD group, we performed hematoxylin–eosin (HE) staining on these two groups of fetal mice. It could be clearly seen that the tissue morphology of the fetal mouse in NTD is not as complete as that of the mice in the normal group, indicating that low-folate-induced mice have apparent neural tube defects (Figure 1B). The serum folate concentrations of 20 female mice in the normal and NTD groups were collected, the data were analyzed, and a boxplot was drawn (Figure 1C, *p* < 0.0001). The results showed that the folic acid concentration in female mice with NTDs was significantly lower than that in the normal group. As the dose of methotrexate (MTX) was increased, embryos showed a more pronounced developmental retardation phenotype, and a significant decrease in the number of normal embryos appeared. When the concentration of MTX was 1.25 mg/kg, the number of normal embryos was 11 (27.5%), the number of developmentally retarded mice was 17 (42.5%), the number of absorbed embryos was 10 (25.0%), and the number of NTD mice was 2 (5.0%). Increasing the dose of MTX to 1.50 mg/kg resulted in a significant reduction in the number of normal embryos, absorbed embryos, and embryos with developmental retardation; there were 10 normal embryos (19.2%), 18 with developmental retardation (34.6%), and 5 absorbed fetuses (9.6%). In contrast, the number of embryos with NTDs significantly increased to 19, accounting for 36.5% of the total number. The radar chart indicates that as the dose of MTX increased to 2.50 mg/kg, there was no NTD embryo phenotype, the number of normal embryos dropped to 4 (6.2%), and the number of absorption and developmentally retarded embryos increased to 15 (23.0%) and 46 (70.8%) (Figure 1D). We established mouse models and performed a proteomic analysis with mass spectroscopy on fetal mouse tissues. The experimental process is shown in Figure 1E. Fetal mice from two groups were taken out at E13.5, and their brain and spine tissues were collected for protein extraction and enzymatic hydrolysis. Each TMT/iTRAQ labeling included two identical collection samples; we used TMT-labeled peptides and adjusted the concentration. The separated peptides, obtained through HPLC, were subjected to LC-MS/MS analysis using a tandem mass spectrometer after being injected into the NSI ion source. Overall, the mouse model of NTDs was induced by low folic acid feeding combined with MTX, and embryonic brain tissue was collected to extract protein and provide an experimental basis for subsequent analysis of proteomic changes.

### 3.2 Protein identification overview

**FIGURE 2:**
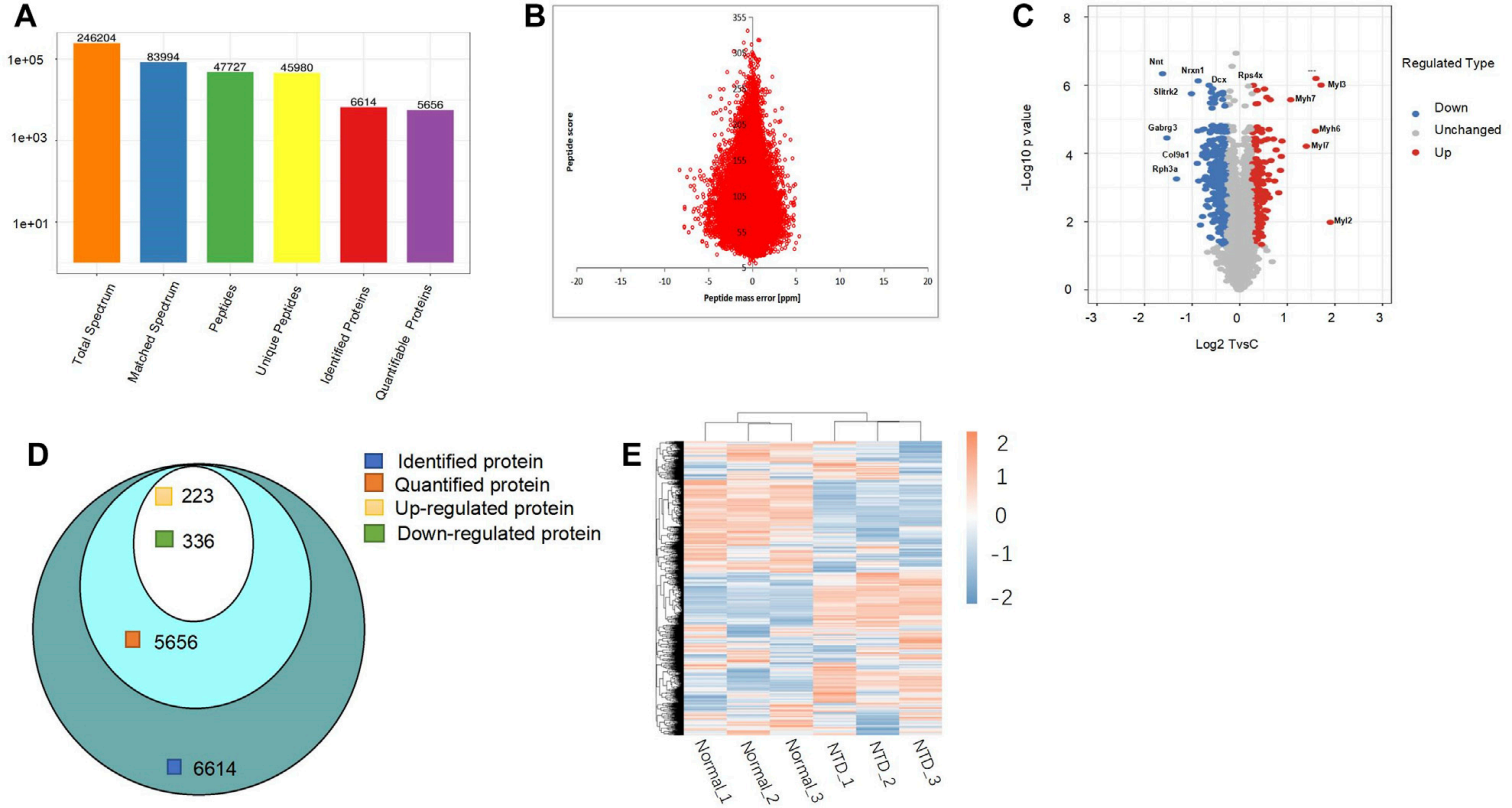
**(A)** Protein identification overview by LC-MS/MS. **(B)** The accuracy distribution of mass spectra using a peptide quantitative analysis diagram. **(C)** Volcano plot for quantification of DEPs. **(D)** Venn diagram of the number of proteins detected by spectral analysis, quantified proteins, and upregulated and downregulated proteins. **(E)** Heatmap of all quantified proteins in the normal group and NTD group. DEPs, differentially expressed proteins; NTDs, neural tube defects.

A total of 246,204 secondary spectra were acquired through mass spectrometry (MS) analysis. Analysis using secondary spectroscopy followed by searching in protein theory databases, and 83,994 matched spectra were obtained. The spectrum utilization rate was 34.1%. Spectrum analysis yielded 47,727 peptides, of which 45,980 were unique; the total number of identified proteins was 6,614, and 5,656 proteins were quantified. The detailed statistics of the data are shown in Figure 2A. Our peptide quantitative analysis is shown in Figure 2B. The distribution of the change and statistical significance of all quantitative proteins is shown in Figure 2C with a volcano plot. The horizontal axis is the value after the Log2 logarithmic transformation of the protein relative quantitative value, and the *p*-value after −Log10 logarithmic transformation is used as the vertical axis. In the volcano plot, the downregulated proteins were Nnt, Nrxn1, Dcx, Slitrk2, Gabrg3, Col9a1, and Rph3a. The upregulated DEPs were Rps4x, Myl3, Myh7, Myh6, Myl7, and Myl2. Among them, downregulation of NRXN1 in mice impairs neurite outgrowth, which in turn leads to neuronal and synaptic damage (Wu et al., 2023). Astrocytes in the CNS were also involved in signal transduction and synapse formation; the downregulation of NRXN1 leads to functional defects of astrocytes and also affects neural tube development (Zeng et al., 2013). Thus, it can be inferred that the development of the neural tube is impacted by the regulation of these proteins. Among the 6,614 proteins detected by spectral analysis, there were 5,656 quantifiable proteins, of which 223 and 336 proteins were significantly upregulated and downregulated, respectively (Figure 2D). A normal diet and a low FA diet were used to establish two mouse models. We divided the samples into three groups: Normal1, Normal2, Normal3 and NTD1, NTD2, and NTD3 and then used these data to draw a heatmap (Figure 2E).

### 3.3 Functional classification of differentially expressed proteins associated with NTDs

**FIGURE 3:**
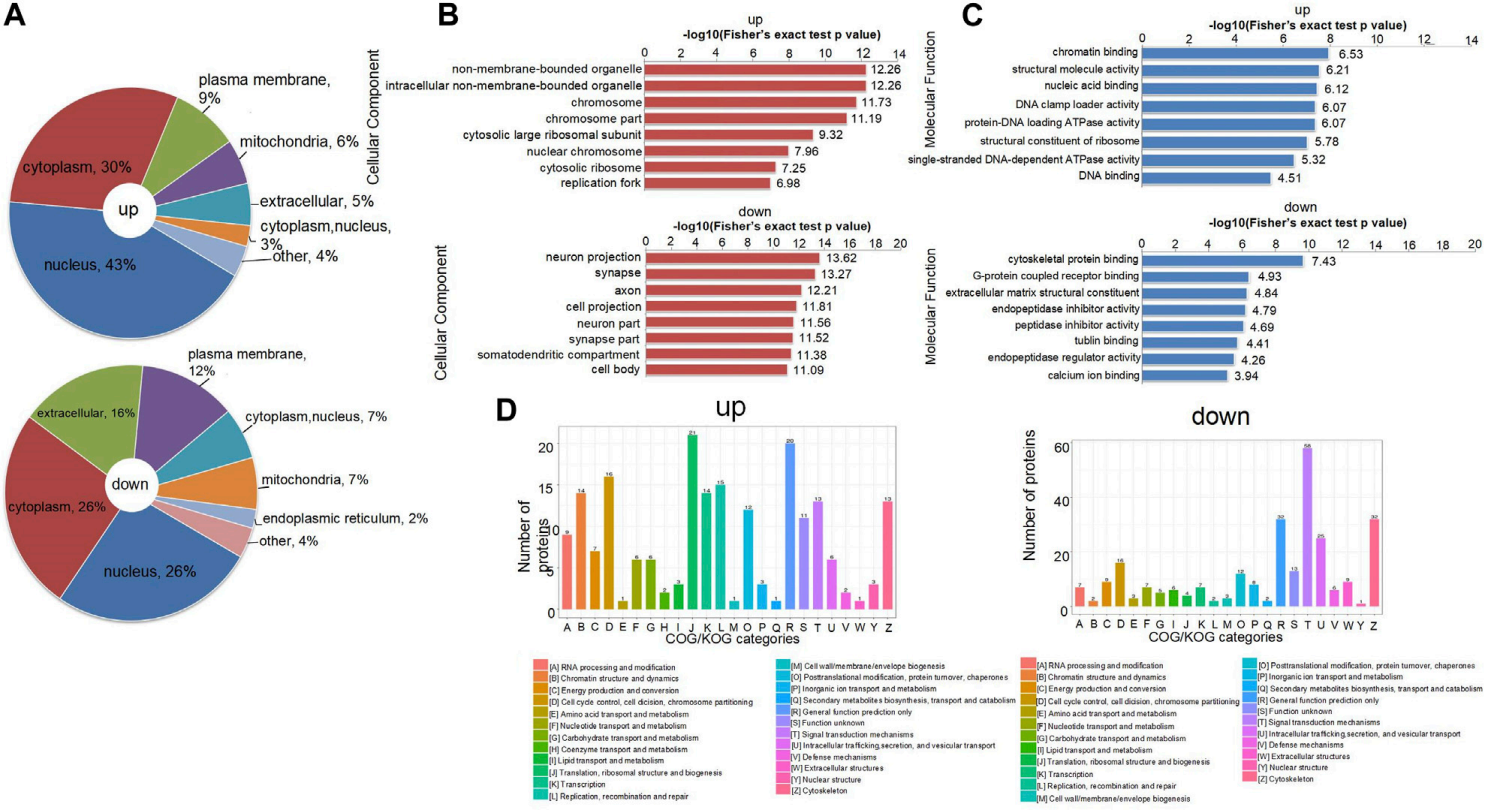
**(A)** Distribution of subcellular structure localization of upregulated and downregulated differentially expressed proteins. **(B)** *p*-value bar chart of upregulated and downregulated DEPs of cellular components in GO terms from Fisher’s exact test. **(C)** *p-*value bar chart of upregulated and downregulated DEPs of molecular function in GO terms from Fisher’s exact test. **(D)** COG/KOG functional classification distribution map of upregulated and downregulated DEPs. GO, gene ontology.

There is a set of specific proteins in each subcell, which provides a relatively independent life activity place for these proteins to perform their functions. Proteins are translated and synthesized in the cytoplasm, guided by protein sorting signals, and then transported to specific subcells to participate in various life activities of the cells. The use of subcellular localization methods to classify proteins into different groups helps reveal the pathogenic proteins associated with NTDs and is critical for the subsequent potential treatments (Puvirajesinghe and Borg, 2015). In this study, we analyzed DEPs by classification and statistics of subcellular structural localization. The highest number of upregulated DEPs were in the nucleus (43%), followed by the cytoplasm (30%) and the plasma membrane (9%). The proportion of DEPs in the mitochondria and extracellular space was similar. The highest number of downregulated DEPs were in the cytoplasm and nucleus (26%), 16% were in the extracellular space, and 12% were in the plasma membrane (Figure 3A). The number of proteins in the cytoplasm, nucleus, and mitochondria was the same (7%), and the number of DEPs was the least in the endoplasmic reticulum (2%).

When synthesizing proteins for functional interpretation, the first step is to combine protein identity with relevant GO terms (Ashburner et al., 2000). These GO terms can be grouped: cellular components, which are the subcellular structures, locations, and parts of the cell; molecular functions, such as binding or catalytic activity; and biological processes, which refer to an ordered combination of molecular functions, such as metabolic processes or embryonic morphogenesis. Because we mainly studied the proteomic analysis of NTDs, the DEPs of cellular composition and molecular function are selected for display (Supplementary Figure S1). These two terms contain several typical type descriptions. The horizontal axis was expressed as the *p*-value after the logarithmic transformation of −Log_10_, which made the data more in line with normal distribution, and then a bar graph was drawn. Through the enrichment analysis of GO terms related to NTDs, we concluded that both chromosomal and large ribosomal subunits have a significant role in the upregulated histogram of cellular composition; in the downregulated histogram, the cellular compositions associated with NTDs are neuron projections, synapses, and axons (Figure 3B). Figure 3C reveals that chromatin binding, nucleic acid binding, and protein-DNA loading ATPase activity through the upregulated bar chart are related to NTDs. DEPs were subjected to homologous classification analysis using clusters of orthologous groups (COGs) of proteins, mainly divided into prokaryotes and eukaryotes, and compared and analyzed through the COG database and KOG database, respectively. In the upregulation chart, D: Chromosome partitioning, cell cycle control, cell division, and J: Biogenesis, translation, and ribosomal structure have the largest number of DEPs. In the downregulation chart, T: Signal transduction mechanisms have the largest number of DEPs (Figure 3D). Taken together, it can be seen that the DEPs in different functional classifications regulate the occurrence of NTDs.

### 3.4 Cluster analysis based on protein domain, GO analysis, and KEGG pathway

**FIGURE 4:**
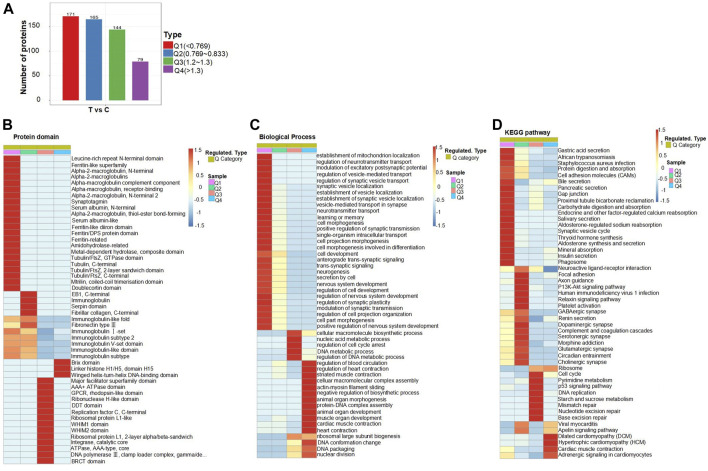
Cluster analysis of DEPs: **(A)** DEPs were divided into Q1–Q4 according to similarities. **(B–D)** Heatmap of cluster analysis based on protein domain, the biological process of GO classification, and KEGG pathway. Cluster analysis heatmap of cellular components and molecular functions of GO classification can be found in Supplementary Figure S2.

According to the magnitude of differential expression (Figure 4A), we categorized the DEPs in the brain tissues of mice treated with folate deficiency into four groups: Q1, Q2, Q3, and Q4; the number of proteins in each group was represented on the vertical axis of the graph. Comparing the normal and treated mice, the number of DEPs was 171, and the multiple of difference is less than 0.769, which we defined as Q1; the multiple of difference for the Q2 group ranged from 0.769 to 0.833, the multiple for Q3 ranged from 1.2 to 1.3; when the multiple of difference exceeded 1.3, the DEPs were classified into the Q4 group. Then, we performed an enrichment analysis of the protein domain, the GO classification, and the KEGG pathway for each Q group. Through enrichment analysis, the enrichment test *p*-value (Fisher’s exact test) could be acquired, and the correlation functions in these four groups were clustered together using a hierarchical clustering method and plotted as a heat map (Figures 4B–D and Supplementary Figure S1). In the heatmap, the horizontal axis displayed the enrichment of four groups, while the vertical axis represented the enrichment of differentially expressed functions related to GO, the protein domain, and the KEGG pathway. Different color blocks correspond to each group of DEPs, and functional descriptions in each heatmap represent different degrees of enrichment. Red represents the strongest enrichment intensity, and the percentage of protein in the functional description corresponding to this group was relatively large. Blue represents weak enrichment intensity, and the percentage of protein in the functional description consistent with this group is small. On the whole, in the heatmap of biological processes, the differential expression of functions related to neural tube development mainly showed higher enrichment intensity in the Q1 group. Moreover, there were also related signaling pathways in the KEGG heat map, such as the PI3K-Akt and p53 signaling pathways, which were mainly enriched in the Q2 and Q3 groups, while ribosomes in GO and KEGG pathways had similar functional enrichment levels in the Q3 and 4 groups.

### 3.5 The PPI network related to NTDs

**FIGURE 5:**
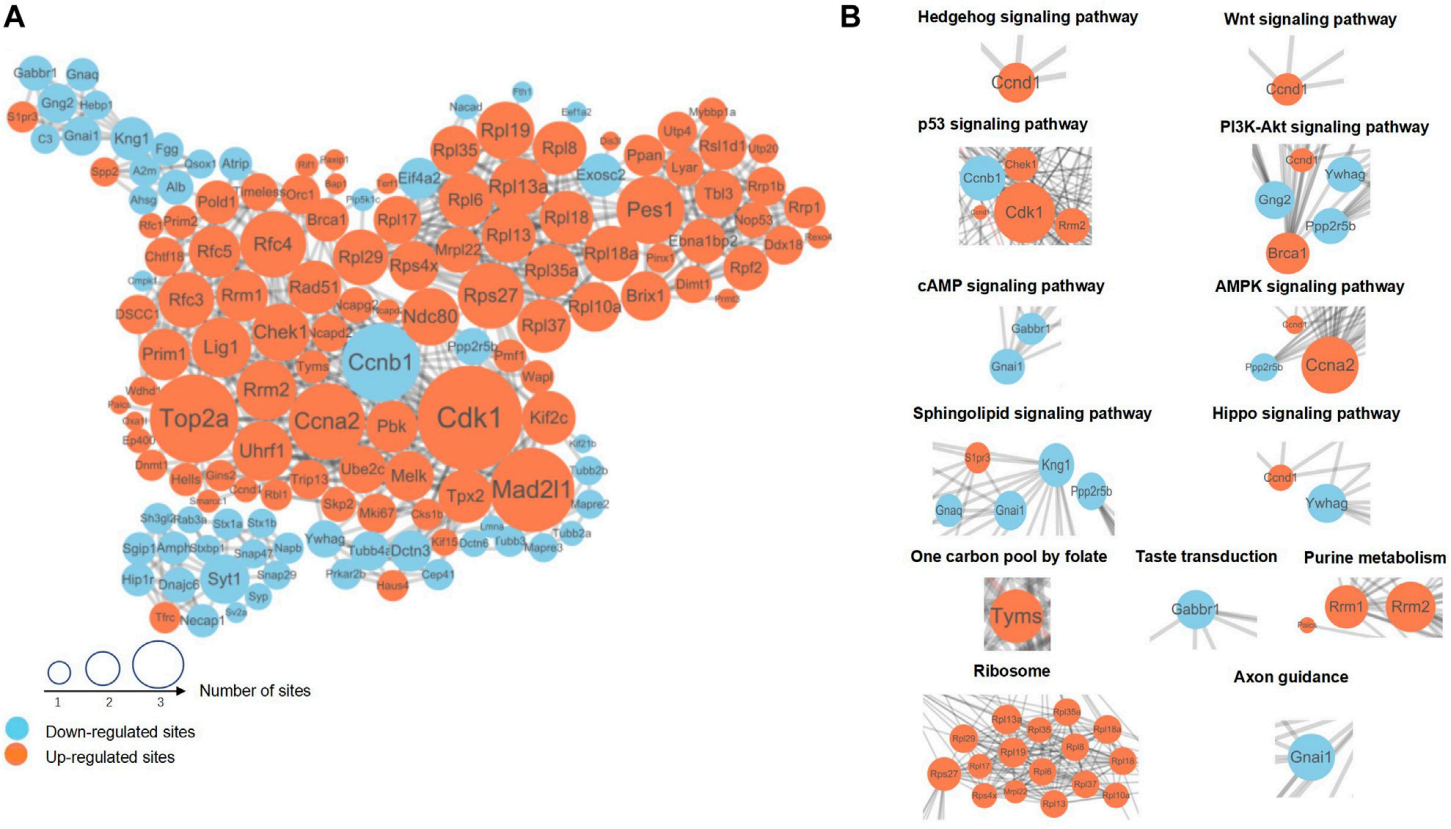
**(A)** The DEPs with the most closely interacting relationships. The size of the sites represents the number of differential proteins and their interacting proteins. Blue indicates downregulated proteins; orange indicates upregulated proteins. **(B)** DEPs and their interacting proteins were associated with NTDs in the PPI network. PPI, Protein–protein interaction.

In order to study the DEPs related to the occurrence of NTDs, we performed a PPI network analysis and identified key proteins with potential effects on the generation of NTDs. After comparing the protein sequences screened by different groups in the cluster analysis with the protein network interaction database, interaction confidence of extracted differential proteins > 0.7, and the PPI network was visualized by using *Cytoscape (v.3.7.1)* (Figure 5A). In this figure, the DEPs are represented by circles, with larger circles indicating more interacting protein sites and suggesting their important roles in the PPI network. Examples of such DEPs include Cdk1, Ccnb1, Ccna2, Rps27, Rrm2, and Kng1. Different colors indicate different differential expression situations, among which blue is downregulated proteins and orange is upregulated proteins. The DEPs in metabolic and signaling pathways related to the generation of NTDs can be found intuitively using the PPI network.

Referring to the proteins of the metabolism and signaling pathways related to NTDs in the KEGG pathway, the interaction between the corresponding proteins in each pathway can be found in Figure 5A. Among them, we selected typical pathways and displayed the differentially expressed protein interaction network in Figure 5B. Hedgehog, Wnt, p53, and Hippo signaling pathways and the one-carbon pool mediated by folate were the main impact pathways of NTDs, which have been reported in many previous studies. In addition to these pathways, a variety of ribosomal proteins were represented in PPI networks, as shown in Figure 5B. DEPs with close interactions in the pathways related to neural tube development can be found using the PPI network diagram. The proteins in these pathways have different regulatory levels during the occurrence of NTDs, which can be used as key biomarkers for subsequent verification.

### 3.6 RPL13 and RPL14 ribosomal proteins could potentially play a role in the occurrence of NTDs

**FIGURE 6:**
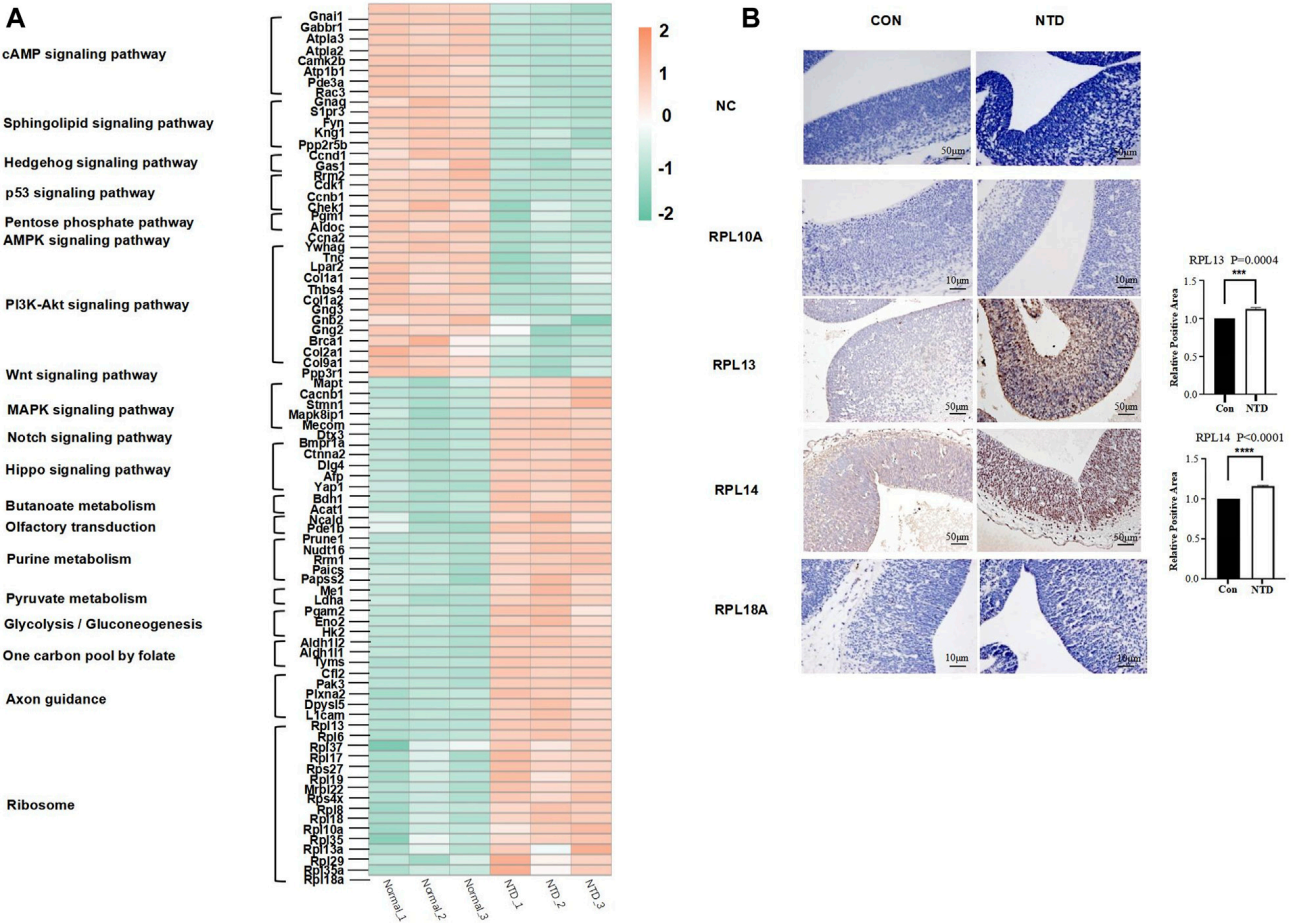
**(A)** Heatmap DEPs were associated with NTDs. **(B)** Immunohistochemical analysis of antibodies to ribosomal proteins (RPL10A, RPL13, RPL14, and RPL18A) was associated with NTDs. The data of RPL13 and RPL14 were mean ± S.D. The *p* values are 2.5241E-06 and 0.0026, respectively; *p < 0.05 by Student’s test.

In this study, we performed a cluster analysis on all DEPs in signaling pathways, metabolic pathways, and ribosomes related to NTDs and generated a heatmap (Figure 6A). We conclude that the DEPs of the cAMP signaling pathway, the Wnt signaling pathway, the sphingolipid signaling pathway, the PI3K-Akt signaling pathway, the Hedgehog signaling pathway, the p53 signaling pathway, the pentose phosphate pathway, and the AMPK signaling pathway had higher expression levels in normal mice. Furthermore, the upregulation of DEPs in the Hippo signaling pathway, the MAPK signaling pathway, the Notch signaling pathway, all metabolic pathways, and the one-carbon pool mediated by folate and ribosomes was observed in all three groups of mice with NTDs. Thus, all of these proteins may have the potential to affect the development of NTDs.

We found 16 ribosomal proteins in the protein interaction network; RPL10A, RPL13, RPL14, and RPL18A are involved in embryogenesis, according to previous studies. Hetman et al. suggested that neurodevelopmental malformations may result from mutations in ribosomal components and trans-acting ribosomal biogenesis factors (Hetman and Slomnicki, 2019). RPL10A is reduced during mesodermal development and leads to a reduction in the translation of mesodermal regulators, including the Wnt pathway, which was found in the ribosomal profiles of mice with a loss of RPL10A function (Genuth et al., 2022). Prior research found that the 60s ribosomal subunit binds to the 48s promoter complex at the mRNA initiation complex, which includes RPL13, RPL14, and RPL18A, and can functionally form the 80s ribosome (Oh et al., 2002). Among them, RPL13 and RPL14 are considered tumor suppressor genes, so changes in the expression of these ribosomal proteins during development may directly alter the ribosome synthesis required for biological development, such as causing delayed closure of the embryonic neural tube or increasing the vulnerability of the embryo to NTDs (Chan et al., 1994). Therefore, we selected four protein antibodies (RPL10A, RPL13, RPL14, RPL18A) from all ribosomal proteins for immunohistochemical staining analysis, as shown in Figure 6B. The control (CON) and NTD groups were set up, and the immunohistochemical staining results of the four protein antibodies were compared with the negative control (NC) group. The staining degree of RPL13 and RPL14 antibodies in the NTD group is deeper than that in the NC group. In addition, the staining reaction of RPL13 and RPL14 antibodies in the CON and NTD groups was statistically significant (*p* < 0.05). This indicated that changes in the expression of RPL13 and RPL14 may lead to the generation of NTDs. Moreover, Western blot analysis was also performed on the brain tissues of fetal mice with three replicates in the normal group and the NTD group at 13.5 days. The results are shown in Supplementary Figure S3. The results from the Western blot are in concordance with IHC.

## 4 Discussion

Folate deficiency contributes to birth malformations (van Gool et al., 2018; Lee and Gleeson, 2020; Kancherla et al., 2022). Supplementation of folic acid in preparation for pregnancy and during early pregnancy greatly reduced the occurrence and recurrence rates of fetal NTDs and malformations (Smithells et al., 1980; Lancet, 1991; Czeizel and Dudas, 1992; Dean et al., 2020). Although NTD-related pathologies in mouse models have been extensively studied from the gene expression perspective (Krupp et al., 2012; Yu et al., 2017; Santander et al., 2018; Murphy et al., 2021) and in-depth protein expression in the mouse brain has also been profiled (Erickson et al., 2021), intensive proteome analysis in low-folate-induced NTD mouse fetal brain tissues is uncommon. We performed a proteomic study in a folate-deficiency-induced mouse brain. In this article, we described an effective mass spectrometry method to identify important proteins in brain tissues that are regulated in response to the folate level and addressed proteomic signatures that are affected by folate in brain tissues. Through this proteomic study, a total of 6,614 proteins were identified, of which 5,656 contained quantitative information. By comparing normal mice with NTD mice, we found that 223 proteins were upregulated and 336 proteins were downregulated. Folate and NTD-related shifts occur in commonly known NTD-related pathways such as the Hedgehog signaling pathway (Murdoch et al., 2010), the Wnt signaling pathway, the P53 signaling pathway (Patterson et al., 2006), the PI3K-Akt signaling pathway (Tian et al., 2022), the cAMP signaling pathway (Shimada and Mukhopadhyay, 2017), the AMPK signaling pathway (Zhang et al., 2021), the sphingolipid signaling pathway (Callihan et al., 2012; Lai et al., 2016), the Hippo signaling pathway (Manderfield et al., 2014; Wang et al., 2016), the one-carbon pool mediated by folate (Copp et al., 2013), axon guidance (Connor et al., 2005), glycolysis and gluconeogenesis (AdamsMJTNEjom, 1984; Wang et al., 2023), purine metabolism (Imbard et al., 2013), and some underappreciated pathways, such as taste transduction and ribosome.

In the past decades, NTD-relevant studies have been done from a transcriptome, metabolomics, and proteomics perspective (Fan et al., 2011; Hansler et al., 2014; Liu et al., 2014; Puvirajesinghe and Borg, 2015; Yu et al., 2017; Wang et al., 2022). In transcriptomic profiles at embryonic days (E) 8.5, 9.5 and 10.5 d in a retinoic acid-induced NTD model, Yu et al., found that genes such as Bmp2, Ascl1, Olig2, Lhx1, Wnt7b, Eomes, Foxp2, Hoxb3, Gpr56, Hap1, Nkx2-1, Sez6l2, Tbx20, Nfib, Cntn1, Dcx, Gpr56, Ngrn, Ddr1, Dctn1, Dnmt3b, Ect2, Map2, Mbnl1, Meis2, Vcan, App, Nova1/2, nSR100/Srrm4, Elavl3/4, Celf3, and Rbfox1 were differentially expressed during mouse neural tube development (Yu et al., 2017). Hansler et al. reported untargeted metabolite profiling in NTD-affected Lrp6 ^−/−^ whole embryos that revealed abnormal methionine regeneration, one-carbon metabolism, and antioxidant activity (Hansler et al., 2014). A recent proteomic study in maternal serum exosomes identified two proteins (coronin1 A and dynamin 2) on gestational days E12 to E18 that were significantly downregulated (Wang et al., 2022). Another proteomics study identified Crpm-4, Hsp-70, and calponin-3 functioning in apoptosis, folding, signal transduction, transcription, and protein synthesis that could be used as a future diagnosis and treatment methods for spina bifida (Fan et al., 2011).

To our knowledge, this is the first proteomic study on a folate-deficiency-induced mouse brain. Because folate deficiency alone does not lead to NTDs, in our previous report, we constructed a mouse model using folate deficiency as a sensitizing agent (Pei et al., 2019). Parental mice treated with an injection of MTX at 1.50 mg/kg had the highest NTD birth rate. When the MTX dose increases to 2.50 mg/kg, most of the embryos are absorbed. Low folate acts as a sensitizing factor. Folate deficiency coupled with some external factors such as gene mutation or drugs could lead to NTDs. The prevalence of NTDs was attributed to genetic, non-genetic, epigenetic, and environmental factors. Environmental factors may influence neural tube closure through a direct effect on metabolism. Folate deficiency leads to delayed embryonic development and growth retardation in early embryonic progression, but alone does not lead to NTDs. Mouse models are largely used to study human diseases because mouse genomes have great similarity to humans (Krupp et al., 2012). A major number of neural tube malformation mouse models were constructed with RA (Yu et al., 2017; Cheng et al., 2019; Wang et al., 2022; Xiaolu et al., 2023). Mouse neural tube closure is a process that happens between embryonic day E8.5 to E10.5 (Copp, 2005; Greene and Copp, 2009). In particular, RA-induced mouse NTD models from transcriptomic points were shown by Juan et al. GO showed that the biological processes of pattern specification, regionalization, and regulation of nitrogen compound metabolic processes were more enriched on E8.5 than on E9.5. Meanwhile, nerve system development, cell communication, cell–cell signaling, and ion transport were more enriched on E9.5 than on E10.5. In line with this, low-folate-induced mouse NTD models from our study showed that cell development, neurogenesis, and nervous system development are highly enriched on E13.5. Furthermore, KEGG in RA-induced mouse NTD models implies that over-activation in the hedgehog MAPK signaling pathway contributes to hyperglycemia-induced congenital NTDs and regulates cell apoptosis during neural tube formation. Likewise, proteins involved in MAPK Hippo signaling pathways are activated in low-folate-induced mouse NTDs.

Folate is the natural form of folic acid, an essential water-soluble vitamin, and is involved in principal metabolic pathways (Imbard et al., 2013). Intake of FA in the periconceptional period greatly reduced NTD occurrences (Lancet, 1991; Czeizel and Dudas, 1992; Berry et al., 1999). Although folate is tightly related to the occurrences of NTDs, folate deficiency alone does not lead to NTDs. Thus, we established an NTD mouse model induced by a folate-deficient diet. This model allows us to study the relationship between folate and the occurrence of NTDs in mice. In response to a low-folate diet, changes related to folate deficiency are apprehended by upregulation in ribosomal RNAs (rRNA). The DEPs, including large subunit ribosomal protein L13e (RPL13), large subunit ribosomal protein L6e (RPL6), large subunit ribosomal protein L18e (RPL18), large subunit ribosomal protein L10Ae (RPL10A), large subunit ribosomal protein L18Ae (RPL18A), large subunit ribosomal protein L13Ae (RPL13A), large subunit ribosomal protein L37e (RPL37), large subunit ribosomal protein L17e (RPL17), small subunit ribosomal protein S27e (RPS27), large subunit ribosomal protein L19e (RPL19), large subunit ribosomal protein L22 (MRPL22), small subunit ribosomal protein S4e (RPS4), large subunit ribosomal protein L8e (RPL8), large subunit ribosomal protein L35e (RPL35), large subunit ribosomal protein L29e (RPL29), large subunit ribosomal protein L35Ae (RPL35A), and large subunit ribosomal protein L18Ae (RPL18A), were found in the PPI network named the ribosome subgroup. In mammals, the ribosome is a 4 MDa structure with two subunits. The 40s subunit is encoded by Rps genes, and the 60s subunit is encoded by the Rpl genes (Moss et al., 2007).

Ribosomes are vital in producing cellular proteins necessary for cell growth in life (McCann and Baserga, 2013). Ribosome biogenesis is a complicated process that requires strict regulation and orchestrated coordination of connected proteins to guarantee appropriate ribosomal function (Verma and González-Rico, 2020). During early forebrain development (E8.5 to E12.5), ribosome biogenesis was downregulated (Chau et al., 2018). We emphasized the upregulation of ribosomal proteins, and immunohistochemistry confirmed the increased ribosomal protein expression of Rpl13 and Rpl14. Similarly, protein expression levels of these two ribosomal proteins in fetal brain tissue of NTD mice were also found to be elevated by Western blot. However, You et al. reported that the transcription level of Rpl10 was reduced at E8.75 in Brpf1 mutant mouse embryos (You et al., 2015). In line with this, a study using golden hamsters by Yu et al. showed upregulation of RPL30 mRNA during normal neurulation and demonstrated extreme inhibition in NTDs (Yu et al., 2009). In contrast, the downregulation of ribosomes through early forebrain development has been proposed by Naomi et al. While the reduction of ribosomal protein and ribosome biogenesis sometimes does not result in the downregulation of ribosomal protein translation, this is possibly because the extra-ribosomal functions of some ribosomal proteins are not limited to translation (Fernández et al., 2014). The function of ribosomal proteins is dynamic and complicated. Further studies are required to study the role of ribosomes in folate deficiency-induced NTDs, and the limitation of this study is that the only fetal brain used was from E13.5. We could design a future study to collect samples from E8.5 to E10.5.

## 5 Conclusion

In conclusion, we applied a novel, cost-effective proteome profile to study the brain and spine in response to folate deficiency. We recognized several protein signatures and gene networks. These recognitions allow us to see folate-related biological pathways and metabolic changes. Three main arrangements appeared: upregulation of ribosome-related changes, upregulation of the one-carbon pool mediated by folate-related changes, and upregulation of purine metabolism in folate deficiency-induced NTDs. We focused on the genes in response to folate deficiency and identified key regulators in the processes to provide scientific evidence for early diagnosis of NTDs.

## Data Availability

The original contributions presented in the study are included in the article/Supplementary Material, further inquiries can be directed to the corresponding author.
