# Supplementary figures and images for: Multiplexing of TMT labeling reveals folate-deficient diet-specific proteome changes in NTDs
Source: Front Cell Dev Biol. 2024 Mar 13;12:1294726. doi: 10.3389/fcell.2024.1294726 (PMC10966907; doi:10.3389/fcell.2024.1294726)

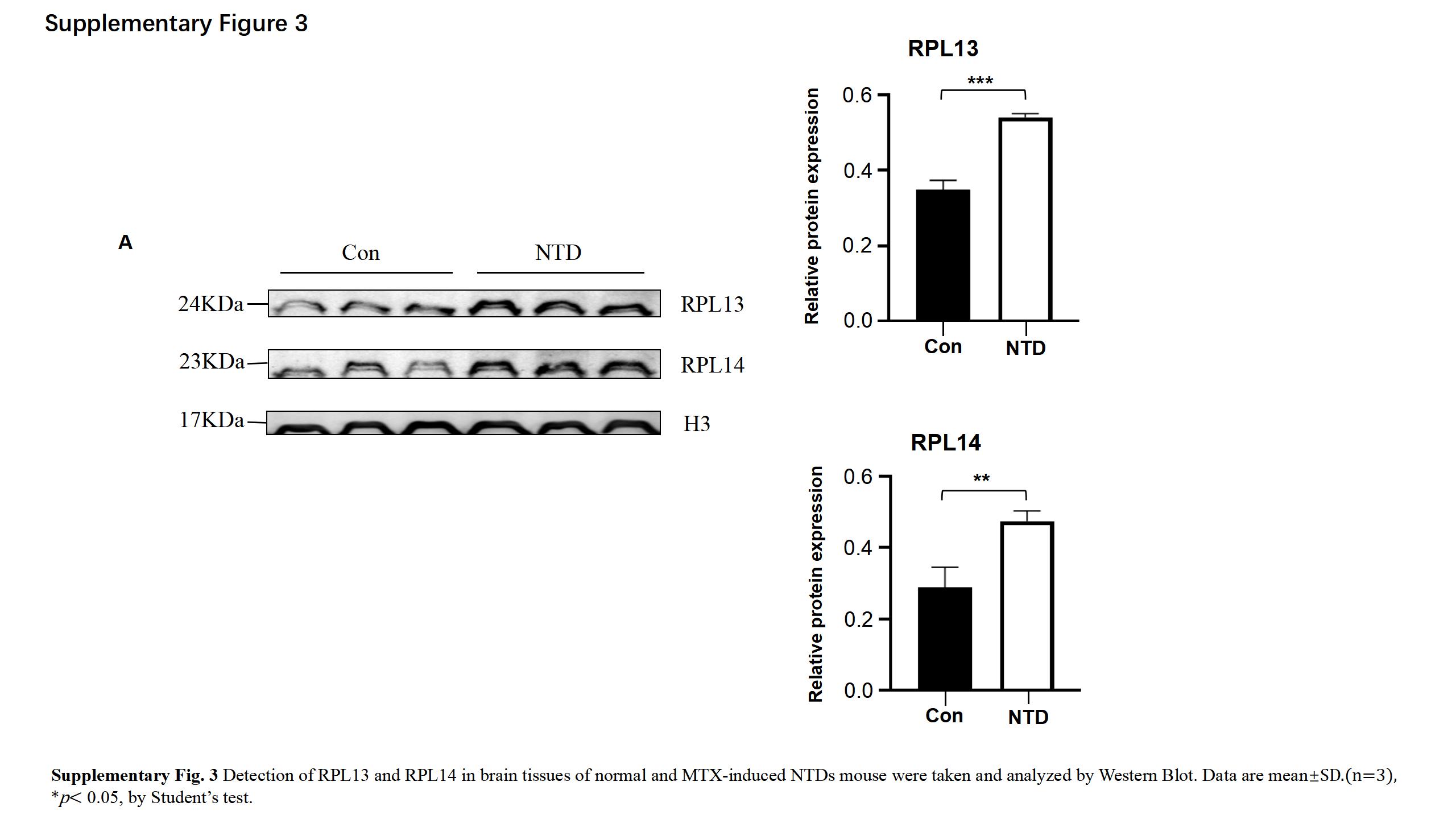

Supplement: Supplementary file 1 [file Image3.TIF]

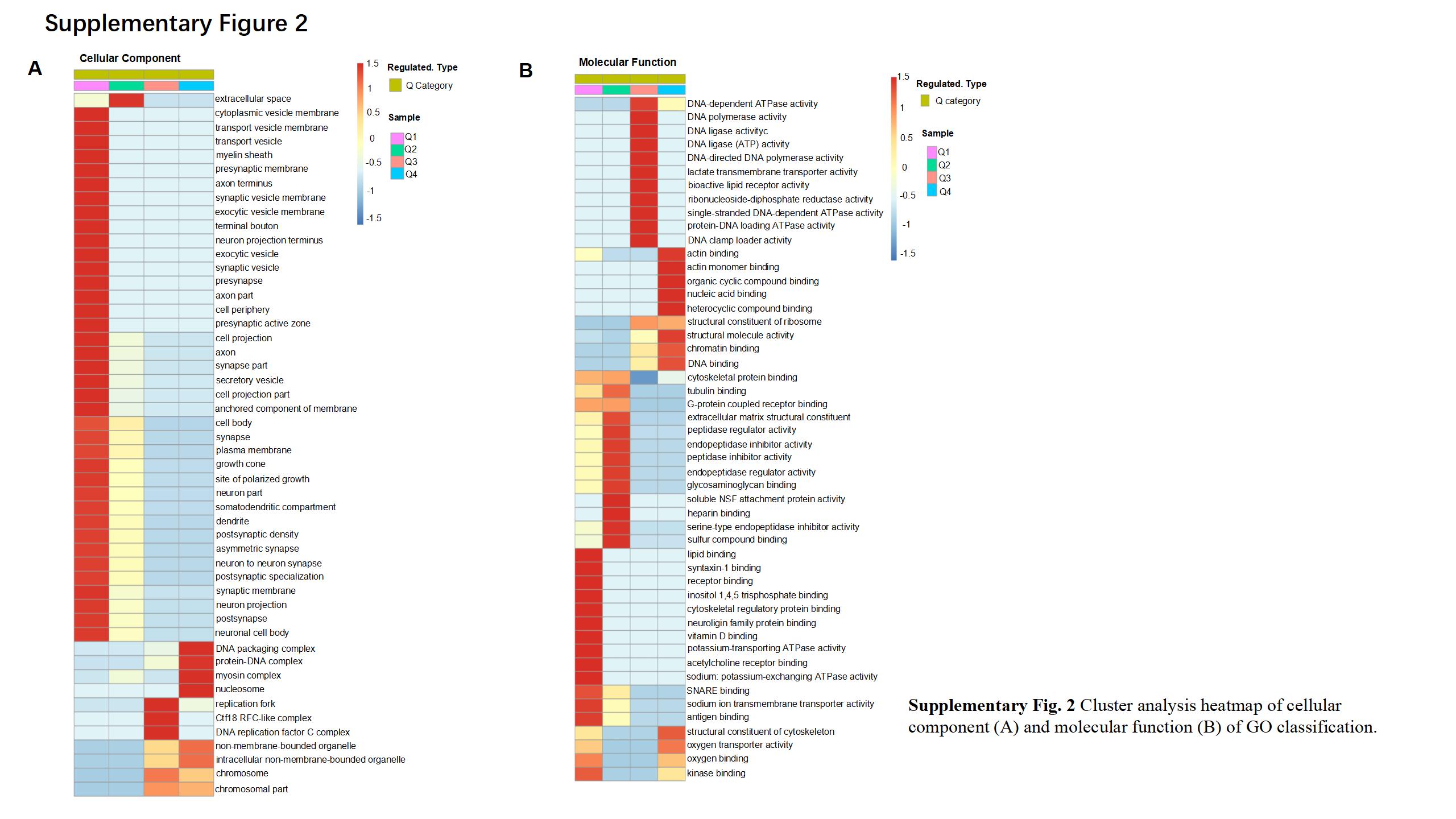

Supplement: Supplementary file 2 [file Image2.TIF]

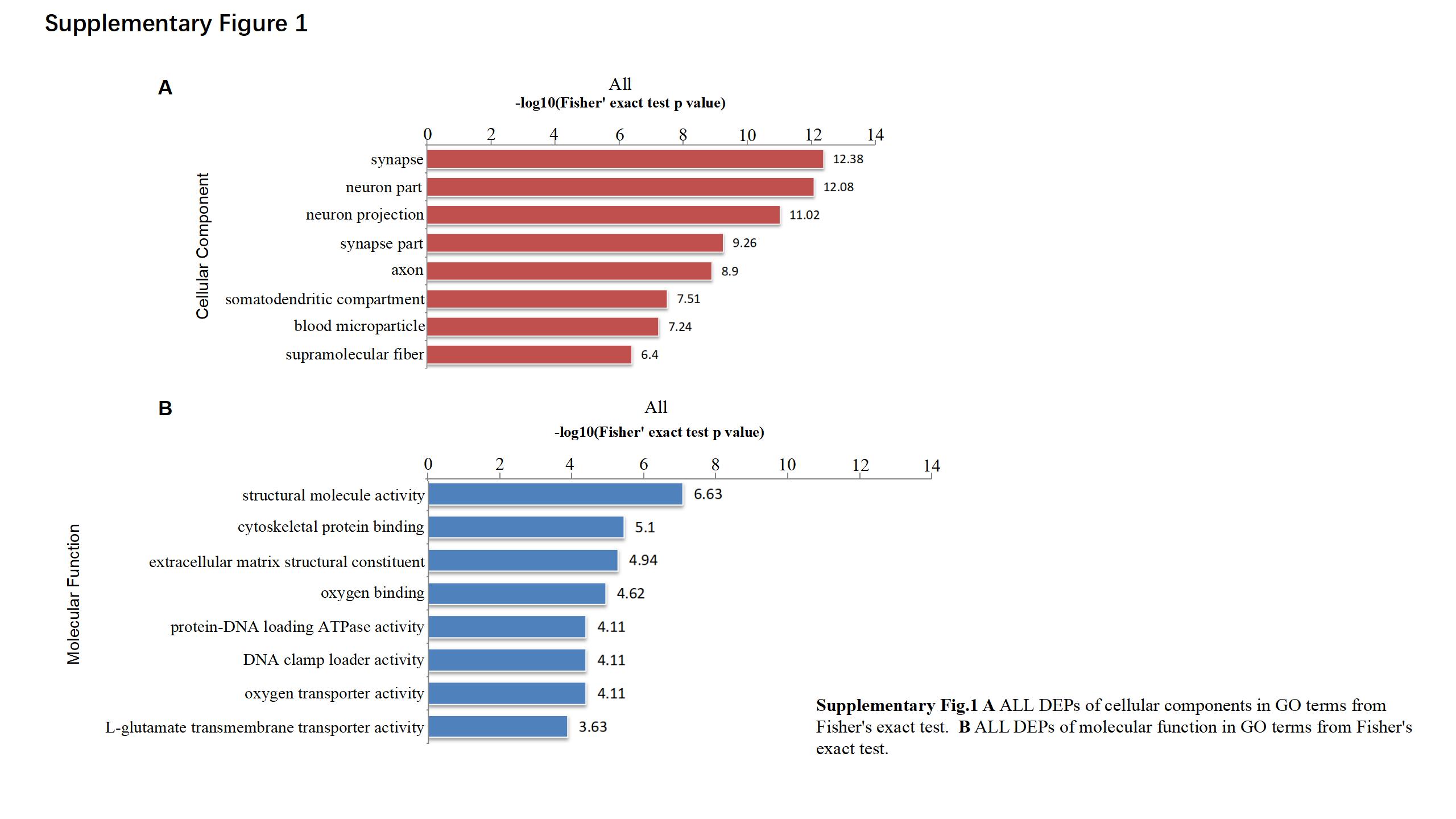

Supplement: Supplementary file 3 [file Image1.TIF]
